# Synthesis of *N*,*N*′-bis(1,5-dimethyl-2-phenyl-1,2-dihydro-3-oxopyrazol-4-yl) sebacamide that ameliorate osteoarthritis symptoms and improve bone marrow matrix structure and cartilage alterations induced by monoiodoacetate in the rat model: “Suggested potent anti-inflammatory agent against COVID-19”

**DOI:** 10.1177/0960327120945779

**Published:** 2020-08-25

**Authors:** MS Refat, RZ Hamza, AMA Adam, HA Saad, AA Gobouri, FA Al-Salmi, T Altalhi, SM El-Megharbel

**Affiliations:** 1 Department of Chemistry, Faculty of Science, 125895 Taif University, Taif, Saudi Arabia; 2 Department of Chemistry, Faculty of Science, Port Said University, Port Said, Egypt; 3 Department of Biology, Faculty of Science, 125895 Taif University, Taif, Saudi Arabia; 4 Department of Zoology, Faculty of Science, 68799 Zagazig University, Zagazig, Egypt; 5 Department of Chemistry, Faculty of Science, 68799 Zagazig University, Zagazig, Egypt

**Keywords:** Anti-inflammatory, antioxidant enzymes, infection, mitochondrial potential

## Abstract

To assess the chondroprotective effect and influence of *N*,*N*′-bis(1,5-dimethyl-2-phenyl-1,2-dihydro-3-oxopyrazol-4-yl) sebacamide (dpdo) that was synthesized through the reaction of phenazone with sebacoyl chloride and screened for its biological activity especially as anti-arthritic and anti-inflammatory agent in a monoiodoacetate (MA)-induced experimental osteoarthritis (OA) model. Thirty male albino rats weighing “190–200 g” were divided randomly into three groups (10 each): control, MA-induced OA, and MA-induced OA + dpdo. In MA-induced OA rat, the tumor necrosis factor alpha, interleukin 6, C-reactive protein, rheumatoid factors, reactive oxygen species, as well as all the mitochondrial markers such as mitochondria membrane potential, swelling mitochondria, cytochrome *c* oxidase (complex IV), and serum oxidative/antioxidant status (malondialdehyde level and activities of myeloperoxidase and xanthine oxidase) are elevated. Also, the activity of succinate dehydrogenase (complex II), levels of ATP, the level of glutathione (GSH), and thiol were markedly diminished in the MA-induced OA group compared to the normal control rats. These findings showed that mitochondrial function is associated with OA pathophysiological alterations and high gene expressions of (IL-6, TNF-a, and IL-1b) and suggests a promising use of dpdo as potential ameliorative agents in the animal model of OA and could act as anti-inflammatory agent in case of severe infection with COVID-19. It is clearly appeared in improving the bone cortex and bone marrow in the treated group with the novel compound in histological and transmission electron microscopic sections which is a very important issue today in fighting severe infections that have significant effects on the blood indices and declining of blood corpuscles like COVID-19, in addition to declining the genotoxicity and inflammation induced by MA in male rats. The novel synthesized compound was highly effective in improving all the above mentioned parameters.

## Introduction

Nowadays the COVID-19 plays a major role in creating a race between chemists and vaccine manufacturers to arrive at a successful treatment for this terrible virus. Chloroquine and, its analogue, hydroxychloroquine are now floating on the surface as a suggestion to be the first line defense for the body against this virus. From the chemical point of view, the small molecule chloroquine directs our attention to synthesize a small molecule similar in size to chloroquine in the hope of success in getting a new vaccine for COVID-19 and to avoid possible side effects of using of large molecules of chloroquine.

Depending on the activities of 4-aminoantipyrine and some of its derivatives in, pharmacology,^1^ medicine,^2^ therefore, some of the 4-aminopyridine derivatives were used to detect the liver diseases^3^ and also, in clinical treatment.^4,5^ Previous studies have been explored to show the effect of 4-aminoantipyrine on human serum albumin and its effect on DNA.^6,7^ Some 4-aminoantipyrine derivatives showed leishmanicidal, antioxidant, and antimicrobial activities.^8^ All of this encouraged us to go through the synthesis of new derivative of 4-aminoantipyrine in the hope of reaching a vaccine that can defeat the virus or even have a promising effect on other microorganisms.

Osteoarthritis (OA) is a painful condition with progressive reduction of articular cartilage, soft tissue swelling at the joints, and bone cyst.^9^ There is no remedy for OA with available therapies, and pain relievers were recommended to alleviate the pain.^10^ The OA pain continues a medical problem, however, animal OA models are being created to enhance the knowledge of OA-related pain processes and reveal new treatment objectives.

There are a lot of OA patients, but no modifying drugs have been developed to treat efficiently OA, and the available medicines are just to relieve symptoms of OA.^11^ Joint replacement is, therefore, the only therapy accessible to OA patients reaching the final phase of OA, indicating the pressing need to develop effective anti-OA medications. One of the unresolved problems in OA pathogenesis is the contribution of the inflammatory response and oxidative stress to the beginning and growth of OA.

During OA pathogenesis, in the joint bodies of OA patients and OA-like animals, a variety of inflammatory mediators, including cytokines, development factors, and prostaglandin E_2_, were noted to be increased.^12^

Moreover, inflammatory reactions along with other threat variables, such as mechanical load and aging, has been revealed to cause oxidative stress by producing nitric oxide (NO), reactive oxygen species (ROS), hydrogen peroxide (H_2_O_2_), superoxide anion, and peroxynitrite, as well as by declining antioxidant enzyme activity.^13^ Although OA has been considered degenerative rather than the intense inflammatory joint disease, the latest trials have shown correlations between OA pathogenesis and inflammatory reactions and oxidative stress.^14–16^ OA is associated with cartilage and chondrocyte of elevated mortality. Cartilage chondrocytes grow in an avascular, low-oxygen setting and maintain integrity in tissue.

Mitochondrial dysfunction can influence a lot of mechanisms involved in cartilage degradation, including oxidative stress, elevated cytokine, and calcification of cartilage matrix, as well as improper chondrocyte biosynthesis, and increased chondrocyte apoptosis.^17^

Chemical-induced models allow studying OA lesions at various phases. The monoiodoacetate (MA)-induced OA model is the most commonly used test to assess the effectiveness of pharmacologic products in the treatment of pain. This model produces a reproducible, solid, and quick pain-like phenotype that can be graded by changing MA dosage.^18,19^ MA injection in rodents intra-articularly recreates OA-like lesions and functional failure which can be analyzed and quantified. MA is a glyceraldehyde-3-phosphatase inhibitor that disrupts cellular glycolysis and ultimately leads to cell death.^20^ MA causes chondrocyte apoptosis, resulting in the degradation of cartilage and subsequent changes in subchondral bone, such as the presence of bone osteophytes.^21^

Nitrogen-containing heterocyclic compounds and their derivatives have historically been invaluable as a source of therapeutic agents. Pyrazole, which has two N-atoms and aromatic character, provides diverse functionality and stereochemical complexity in five-membered ring structure.^22^

In the past decade, studies have reported different data on different pyrazole derivatives and their unnumbered physiological and pharmacological activities. These studies attempted to reveal the wide range of drug-like properties of pyrazole derivatives along with their structure–activity relationships to create opportunities to harness the full potentials of these compounds.^22^ Here, we synthesize one of the pyrazole derivatives and demonstrate that this class of compounds can be targeted for the discovery of new drugs and can be readily prepared owing to recent advances in synthetic medicinal chemistry especially against infection of COVID-19.

Many pyrazole derivatives are acknowledged to possess a wide range of antibacterial bioactivities^23^ and act as DNA gyrase inhibitors, and it is known that DNA gyrase is an essential bacterial enzyme involved in the vital processes of DNA replication, transcription, and recombination. It belongs to a class of enzymes known as topoisomerases, which are involved in the control of topological transitions of DNA. This enzyme possesses a unique ability to catalyze the ATP-dependent negative supercoiling of double-stranded closed circular DNA.^24^

Inflammation is a multifactorial process that reflects the responses of the organisms to various stimuli and is related to many disorders such as pain, redness of skin, swelling, and loss of function in the case of acute inflammation; chronic inflammation may lead to arthritis, psoriasis, and asthma.^25^ Therefore, the role of *N*,*N*′-bis(1,5-dimethyl-2-phenyl-1,2-dihydro-3-oxopyrazol-4-yl) sebacamide (dpdo) on OA symptoms induced by MA was studied in detail at mitochondrial dysfunction, oxidative stress, histological, genotoxicity, and electron microscope levels.

## Materials and methods

### Chemistry

All chemicals used were supplied by Sigma (New York City, New York, USA). Electrothermal IA9100 Standard Digital Melting Point Apparatus, Cole-Parmer (Vernon Hills, IL, USA). Infrared spectra were examined on the ATRAlpha FTIR spectrophotometer. Proton nuclear magnetic resonance (^1^H NMR) and carbon-13 nuclear magnetic resonance (^13^C NMR) spectra were examined on a Bruker AC-400 MHz apparatus (Bruker Corporation, USA). Chemical shifts were expressed as (ppm) relative to internal standard tetramethylsilane (TMS), and dimethyl sulfoxide (DMSO)-*d_6_* was used as the solvent, and in ^13^C NMR, the solvent was DMSO mixture. The elemental analysis of carbon, hydrogen and nitrogen (CHN) analyses and biological activity were achieved in Cairo University at Micro-Analytical Center.

#### N,N*′*-bis(1,5-dimethyl-2-phenyl-1,2-dihydro-3-oxopyrazol-4-yl) sebacamide (dpdo)

Sebacoyl chloride (0.001 mol, 0.24 g), 4-aminoantipyrine (0.001 mol, 0.203 g), and drops of trimethylamine (TMA) were dissolved in N,N,-dimethylformamide DMF (10 mL), and the mixture was refluxed for 6 h (TLC, *R*_F_ = 0.65, eluent: CH_2_Cl_2_/methanol, 9:1). The precipitate obtained was crystallized from methanol solvent (MeOH) to give yellow crystals. Yield, 87%, melting point (m.p.) 184–186°C. Infrared spectra (IR), 3210 cm^−1^ (NH), 2951 cm^−1^ (Ar–H), 2850 cm^−1^ (Aliphatic-H), and 1688–1621 cm^−1^ (C=O and C=N). ^1^H NMR (DMSO-*d_6_*, 400 MHz): *δ* = 1.07 (m, 4H, 2CH_2_, CH_2_C*H*_2_CH_2_CH_2_CONH), 1.10 (m, 4H, 2CH_2_, C*H*_2_CH_2_CH_2_CH_2_CONH), 1.68 (m, 4H, 2CH_2_, CH_2_CH_2_C*H*_2_CH_2_CONH), 2.00 (t, 4H, 2CH_2_, CH_2_CH_2_CH_2_C*H*_2_CONH), 2.28 (s, 6H, 2CH_3_, pyrazole-3-CH_3_), 3.41 (s, 6H, 2CH_3_, pyrazole-*N*-CH_3_), 6.90 (dd, 4H, *N*-Ph-(*H*)_meta_), 7.63 (d, 2H, *J* = 8.4, *N*-Ph-(*H*)_para_), 8.04 (m, 4H, *J* = 7.6, *N*-Ph-(*H*)_ortho_), and 9.30 (s, 2H, N*H*CO). ^13^C NMR (DMSO-*d_6_*, 100 MHz): *δ* = 14.06 (CH_3_), 23.10 (CH_2_CH_2_ *C*H_2_CH_2_CONH), 25.80 (CH_2_ *C*H_2_CH_2_CH_2_CONH), 30.19 (*C*H_2_CH_2_CH_2_CH_2_CONH), 30.77 (*N*-*C*H_3_), 35.78 (CH_2_CH_2_CH_2_ *C*H_2_CONH), 103.78 (pyrazole *C*_4_), 127.95 (phenyl group *C*_2_, *C*_6_), 128.98 (phenyl group *C*_3_, *C*_5_), 130.29 (phenyl group *C*_4_), 133.78 (phenyl group *C*_1_), 142.88 (pyrazole *C*_3_), 162.31 (pyrazole *C*=O), and 177.66 (NH-*C*=O).

### Induction of OA

MA solution in sterile saline (0.9% NaCl) at the required levels was freshly prepared on the day of injection. Once the animal was under anesthesia, the injection location was stabilized in the region surrounding the knee joint.

### Animals and experimental protocol

Animal experiments were conducted following the approval of Zagazig University Animal Ethical Committee (No. Zu-IACUC/1/F/130/2019). The experimental animals were healthy, adult male albino rats, weighing 190–200 g and brought from the Faculty of Pharmacy at Zagazig University, Egypt. The animals were housed with food and water ad libitum under a 12-h light and 12-h dark cycle.

Thirty male rats were divided into five groups of eight animals each. The healthy control group was treated with saline solution (1 mL) (vehicle). The second positive control of OA group was injected by 1 mg of MA in 30 µL of sterile saline in the intra-articular knee joint,^26^ through the knee’s patellar ligament, using a small sharp needle, under anesthesia with sodium thiopental (40 mg kg^−1^). No intervention was carried out in any of the animals after the injection and during the experimental period. There was no use of analgesics or anti-inflammatory drugs. The third group of osteoarthritic rats was injected with MA as previously described and then treated orally with dpdo at a dose of (50 mg kg^−1^).^27^ The experimental period lasted for 45 successive days.

### Determination of pro-inflammatory biomarkers

Blood samples were collected for the determination of serum pro-inflammatory biomarkers at the end of the experiment. Serum levels of tumor necrosis factor alpha (TNF-α) and interleukin-6 (IL-6) were measured according to Cat. Nos R6365 and RB1829, respectively (enzyme-linked immunosorbent assay (ELISA) kit, BIOTANG INC., Lexington, Massachusetts, USA) as recommended by the manufacturer.

### Estimation of inflammation biomarkers

C-reactive protein (CRP) was determined by the method of Wener et al.^28^ using ELISA kit (SEA821). Rheumatoid factors (RF) detection was based on the ability of rheumatoid arthritis sera to agglutinate.^29^ It was determined by using an immunodiagnostic ELISA kit, Biocompare Company, The Buyer’s Guide for Life Scientists (California, USA).

### Mitochondrial function

#### Evaluation of succinate dehydrogenase (complex II) activity

Metabolic viability-based assays using tetrazolium salts like 3-(4,5-Dimethylthiazol 2-yl)-2,5-diphenyltetrazolium bromide (MTT) is used for measuring the mitochondrial metabolic rate and indirectly reflect the viable cell numbers. The mitochondrial succinate dehydrogenase (SDH) activity was evaluated using molecular probe MTT. Mitochondrial suspensions from the knee joints were incubated with MTT probe (0.4% w/v) for 30 min at 30°C. Then, 100 µL of DMSO was used to dissolve the formazan crystals. The total amount of formazan produced upon MTT reduction is directly proportional to the number of viable cells. Finally, the absorbance was measured using an ELISA reader at 570 nm (Tecan, Rainbow Thermo, Austria).^30^

#### Mitochondrial ROS assay

The mitochondrial ROS generation was evaluated using the dichlorodihydrofluorescein diacetate (DCFH-DA) probe. Mitochondrial suspensions from the knee joints were incubated with the DCFH-DA probe at a final concentration of 10 μM for 30 min at 30°C. Finally, the fluorescence intensity of dichlorofluorescein was measured using the Shimadzu RF-5000 U fluorescence spectrophotometer (Kyoto, Japan; *λ*_Ex_ = 488 nm and *λ*_Em_ = 527 nm). An increase in fluorescence intensity indicates an increase in the generation of ROS due to exposure to inflammation of OA.^31^

#### Mitochondria membrane potential (ΔΨ_m_) assay

The mitochondria of the knee joints were isolated from all groups. Briefly, the mitochondrial ROS generation was evaluated using rhodamine 123 (Rh123) probe at a final concentration of 10 μM for 30 min at 30°C. Finally, the collapse of mitochondria membrane potential (MMP) was evaluated using Shimadzu RF-5000 U fluorescence spectrophotometer (*λ*_Ex_ = 490 nm and *λ*_Em_ = 535 nm). An increase in fluorescence intensity indicates an increase in the collapse of MMP.^32^

#### Evaluation of swelling on mitochondria

Briefly, swelling on mitochondria in sizes of 10 and 100 nm (pure and impure) was measured using an ELISA reader (Tecan, Rainbow Thermo) at 540 nm. A decrease in absorbance indicates an increase in mitochondria swelling.^33^

#### Cytochrome c oxidase release assay

Evaluation of cytochrome *c* release (complex IV) that referred to mitochondrial volume was measured (Quantikine Rat/Mouse cytochrome *c* Immunoassay kit, R&D Systems (ab239711), Inc., Minneapolis, Minnesota, USA).

#### ATP content assay

The ATP content was detected using the luciferase enzyme. The intensity was evaluated using Sirius tube luminometer (Berthold Detection System, Germany). ATP content was expressed as nanomoles per milligram protein.^34^

#### GSH content assay

GSH content was determined for isolated mitochondria using 5,5′-dithio-bis(2-nitrobenzoic acid) (DTNB) as the indicator and spectrophotometer (UV-1601 PC, Shimadzu). The mitochondrial suspension was added into 0.1 mol L^−1^ of phosphate buffers and 0.04% of DTNB in a total volume of 3.0 mL (pH 7.4). The developed yellow color was read at 412 nm. GSH content was expressed as micrograms per milligram protein.^35^

#### Lipid peroxidation assay

The malondialdehyde (MDA) content was evaluated to measure lipid peroxidation (LPO). The level of MDA was measured in the supernatant at 532 nm using an ELISA reader (Tecan, Rainbow Thermo). Furthermore, MDA content was expressed as micrograms per milligram protein. An increase in MDA content indicates an increase in the LPO level.^36,37^

#### Myeloperoxidase and xanthine oxidase activities

Myeloperoxidase (MPO) and xanthine oxidase (XO) were detected spectrophotometrically according to Suzuki et al.^36^ and Litwack et al.,^37^ respectively.

#### Total thiols level estimation

It was dogged by the method of Miao-Lin H,^38^ and was presented as millimoles per gram tissue.

### Transmission electron microscope study

Knee joint specimens were dissected, and transmission electron microscopic sections were obtained after fixation in 2.5% glutaraldehyde and then embedded in resin.^39,40^ Tissue blocks were cut serially into ultrathin (0.07 mm) sections, which were stained with uranyl acetate at 4°C for 2 h and lead citrate at 4°C for 20 min. Sections were subsequently observed under transmission electron microscope (JEOL, JSM-5600LV, Japan, magnification, 12,000×, Faculty of Agriculture, Mansoura University, Egypt).

### Histological examination

Bone marrow portions were fixed in about “10% neutral buffer formalin” and then other processing were carried out for preparation of histological slide and further examination and imaging.

### Fluorescent detection of mitochondrial membrane potential

Animals after they were euthanized, hepatic tissues were washed thoroughly and rinsed with ice. They were gently blotted between the folds of a filter paper and weighed in an analytical balance. Ten percent of homogenate was prepared in 0.05-M phosphate buffer (pH 7) using a polytron homogenizer at 40°C according to manual instructions. Fluorescent kit was measured and quantified by fluorometer microplate reader (Thermo Fisher Scientific Oy, Vantaa, Finland). Mitochondrial membrane potential was expressed as JC-1 fluorescence ratio (J-aggregates: J-monomers).

### RNA isolation and quantitative reverse transcription polymerase chain reaction

Gene expressions of knee joint specimens were examined using polymerase chain reaction (PCR) (Table 1). Total RNA was isolated by using TRIzol reagent.

**Table 1.** Primer sequence for PCR amplification.

Gene	Primer sequence	Accession no.	Length (bp)
IL-6	Forward: 5′-TCCTACCCCAACTTCCAATGCTC-3′ Reverse: 5′-TTGGATGGTCTTGGTCCTTAGCC-3′	E02522	79 (Kapoor et al.^41^)
IL-1β	Forward: 5′-CACCTCTCAAGCAGAGCACAG-3′ Reverse: 5′-GGGTTCCATGGTGAAGTCAAC-3′	M98820	111 (Kapoor et al.^41^)
TNF-α	Forward: 5AAATGGGCTCCCTCTCATCAGTTC-3′ Reverse: 5′-TCTGCTTGGTGGTTTGCTACGAC-3′	X66539	79 (Kapoor et al.^41^)

PCR: polymerase chain reaction; IL: interleukin; TNF-α: tumor necrosis factor alpha.

### Real time-PCR

Cytokine transcript levels of IL-1β and IL-6 in the knee joint tissues of male rats were measured using a reverse transcriptase (RT)-PCR technique. Total RNA was extracted from the pancreas by using the iScript™ RT-qPCR.

### Statistical analysis

For statistical calculations, the SPSS 22.0 software program was used. Data were presented as mean ± standard error. Differences between groups were assessed through a one-way analysis of variance followed by Duncan post hoc test (*p <* 0.05).

## Results

### Synthesis of dpdo

**Figure 1.**
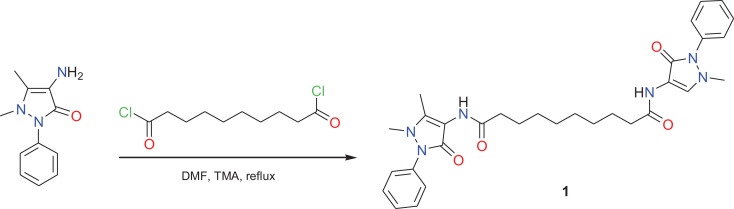
Synthesis of bis sebacamide derivative.

**Figure 2.**
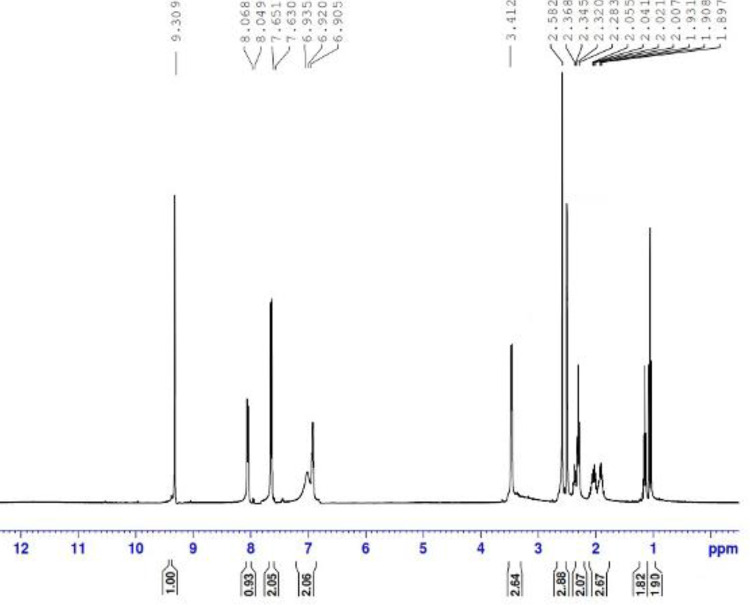
^1^H NMR spectrum of dpdo compound. dpdo: *N*,*N*′-bis(1,5-dimethyl-2-phenyl-1,2-dihydro-3-oxopyrazol-4-yl) sebacamide.

**Figure 3.**
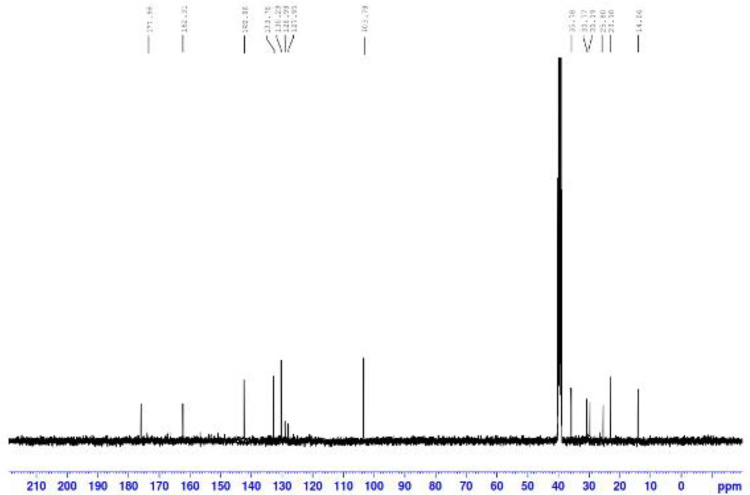
^13^C NMR spectrum of dpdo compound. dpdo: *N*,*N*′-bis(1,5-dimethyl-2-phenyl-1,2-dihydro-3-oxopyrazol-4-yl) sebacamide.

**Figure 4.**
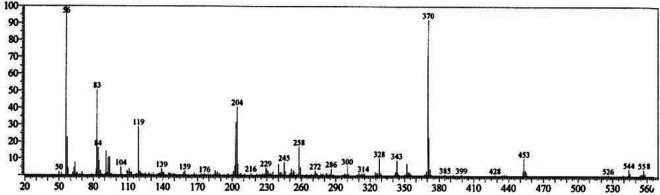
Mass spectrum of dpdo compound. dpdo: *N*,*N*′-bis(1,5-dimethyl-2-phenyl-1,2-dihydro-3-oxopyrazol-4-yl) sebacamide.

**Figure 5.**
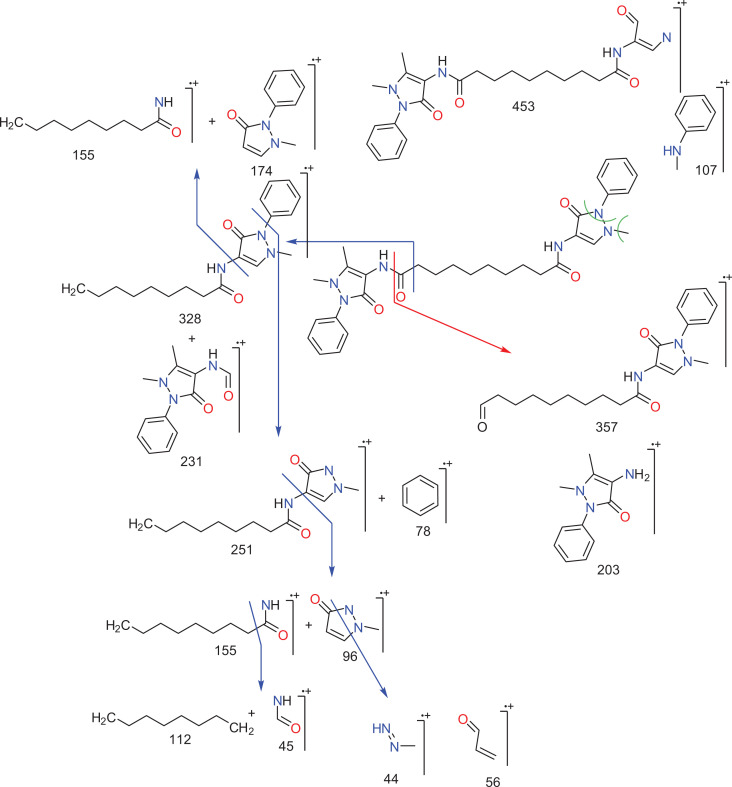
Mass fragmentations of dpdo compound. dpdo: *N*,*N*′-bis(1,5-dimethyl-2-phenyl-1,2-dihydro-3-oxopyrazol-4-yl) sebacamide.

Sebacoyl chloride is, usually, used for the synthesis of a *bis* compounds derivative. Sebacoyl chloride reacted with phenazone in boiling DMF containing TMA as a catalyst to give the sebacamide derivative 1 (Figure 1). The structure of the resulting compound is confirmed from its spectral evidence, ^1^H NMR,^13^C NMR, IR, mass spectra, and analytical data. The IR showed the presence of two carbonyl bands for phenazone and sebacamide moieties. Also showed sharp band for the NH group of the amide group. The ^1^H NMR (Figure 2) also supported the structure conformation by signals for the four CH_2_ and two CH_3_ groups at the range *δ* = 1.07–3.41 ppm. The ^13^C NMR (Figure 3) showed six *sp* ^3^ signals due to the four CH_2_ groups of sebacamide chain at 23.10, 25.80, 30.19, and 35.78 and two signals due to two CH_3_ groups at 14.06 ppm and 30.77 ppm, respectively. Mass spectrum of the synthesized dpdo compound is shown in Figure 4 and its fragmentation assignments are discussed in Figure 5, as *m/z* (int. %): 558 (4.8), 544 (5.5), 526 (2.5), 453 (11.0), 370 (95), 258 (45), 204 (42), 119(33), 83 (52), and 56 (100). Anal. Calcd for C_31_H_38_N_6_O_4_ (558.67): C, 66.65; H, 6.86; and N, 15.04; Found: C, 66.43; H, 6.69; N, 14.94. *m*/*z*: 558.30 (100.0%), 559.30 (34.1%), 560.30 (7.1%), and 559.29 (2.2%).

### IL-6, TNF-α, and CRP levels in the serum

**Figure 6.**
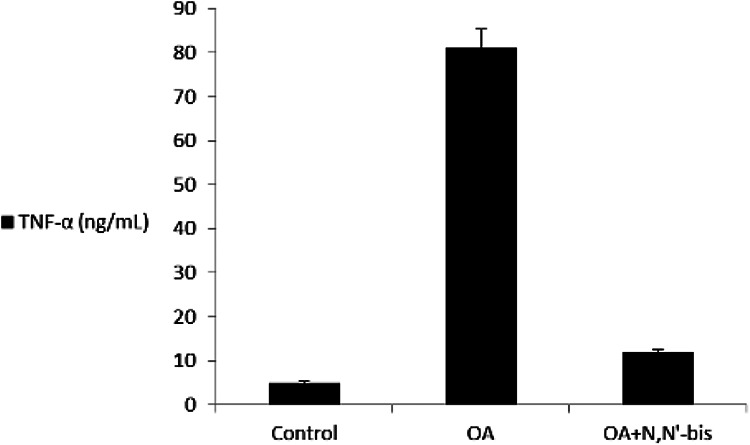
The efficacy of dpdo compound against TNF-α in the rat model of OA induced by MA. The data are presented as mean ± SE. dpdo: *N*,*N*′-bis(1,5-dimethyl-2-phenyl-1,2-dihydro-3-oxopyrazol-4-yl) sebacamide; TNF-α: tumor necrosis factor alpha; OA: osteoarthritis; MA: monoiodoacetate; SE: standard error.

**Figure 7.**
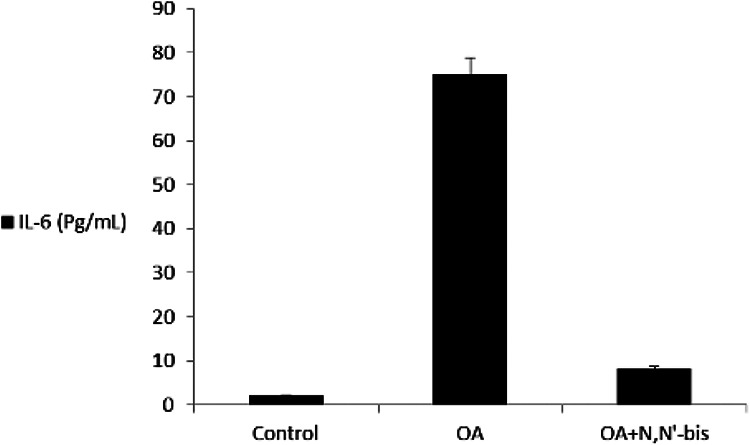
The efficacy of dpdo compound against IL-6 in the rat model of OA induced by MA. The data are presented as mean ± SE. IL-6: interleukin-6; dpdo: *N*,*N*′-bis(1,5-dimethyl-2-phenyl-1,2-dihydro-3-oxopyrazol-4-yl) sebacamide; IL-6: interleukin-6; OA: osteoarthritis; MA: monoiodoacetate; SE: standard error.

**Figure 8.**
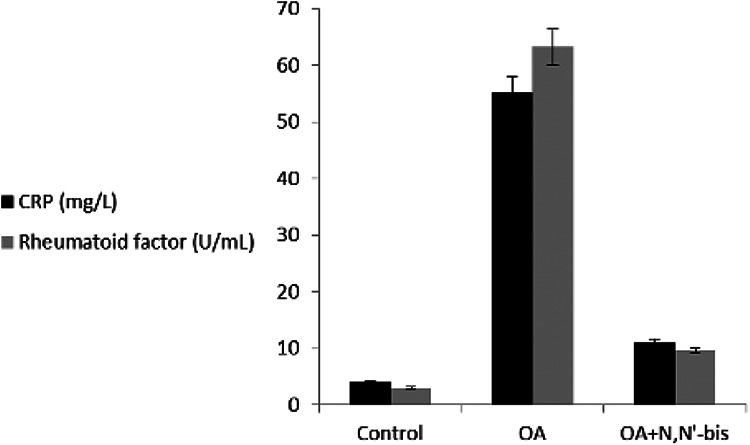
The efficacy of dpdo compound against CRP and rheumatoid factor in the rat model of OA induced by MA. The data are presented as mean ± SE. dpdo: *N*,*N*′-bis(1,5-dimethyl-2-phenyl-1,2-dihydro-3-oxopyrazol-4-yl) sebacamide; CRP: C-reactive protein; OA: osteoarthritis; MA: monoiodoacetate; SE: standard error.

The levels of TNF-α and IL-6 in the serum were detected on day 45 after injection of MA (Figures 6 and 7). The level of these cytokines was markedly increased in the OA group compared with the healthy control animals. The compound dpdo suppressed the TNF-α level as compared to the OA group. Serum CRP level in control rats at baseline was 4.02 ± 0.7 mg L^−1^, with a significant difference by 13.0-fold in case of OA-rats (Figure 8). Importantly, there was a more significant decline in CRP level in rats treated with dpdo in combination (92.7%) compared to the positive control.

### Rheumatoid factors, succinate dehydrogenase, reactive oxygen species, and mitochondria membrane potential

**Figure 9.**
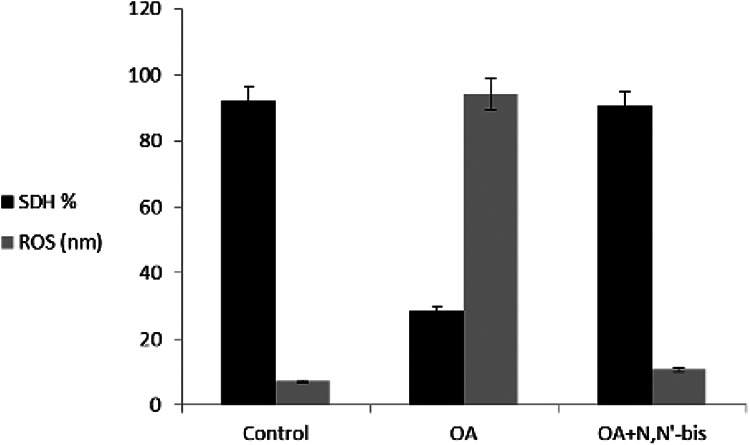
The efficacy of dpdo compound against SDH and ROS in the rat model of OA induced by MA. The data are presented as mean ± SE. dpdo: *N*,*N*′-bis(1,5-dimethyl-2-phenyl-1,2-dihydro-3-oxopyrazol-4-yl) sebacamide; SDH: succinate dehydrogenase; ROS: reactive oxygen species; OA: osteoarthritis; MA: monoiodoacetate; SE: standard error.

**Figure 10.**
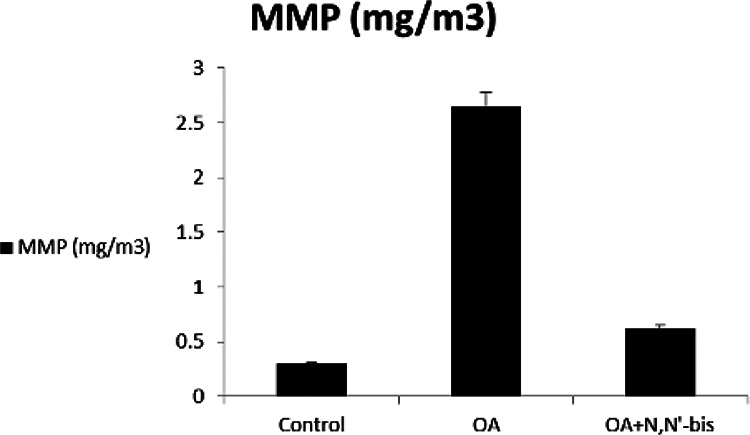
The efficacy of dpdo compound against MMP in the rat model of OA induced by MA. The data are presented as mean ± SE. dpdo: *N*,*N*′-bis(1,5-dimethyl-2-phenyl-1,2-dihydro-3-oxopyrazol-4-yl) sebacamide; MMP: mitochondria membrane potential; OA: osteoarthritis; MA: monoiodoacetate; SE: standard error.

**Figure 11.**
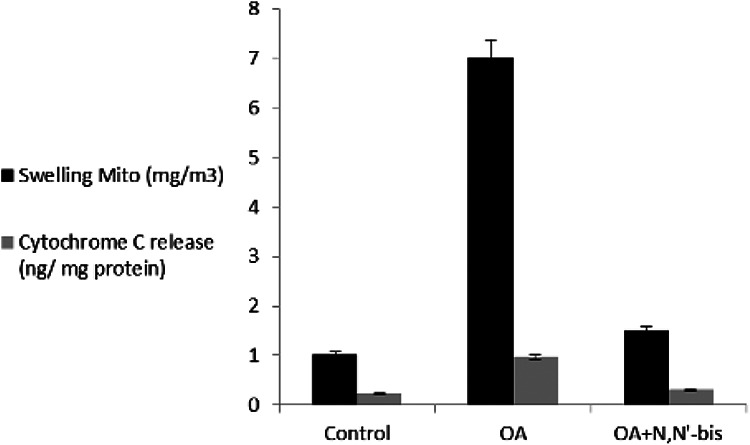
The efficacy of dpdo compound against swelling mitochondria and cytochrome *c* release in the rat model of OA induced by MA. The data are presented as mean ± SE. dpdo: *N*,*N*′-bis(1,5-dimethyl-2-phenyl-1,2-dihydro-3-oxopyrazol-4-yl) sebacamide; OA: osteoarthritis; MA: monoiodoacetate; SE: standard error.

**Figure 12.**
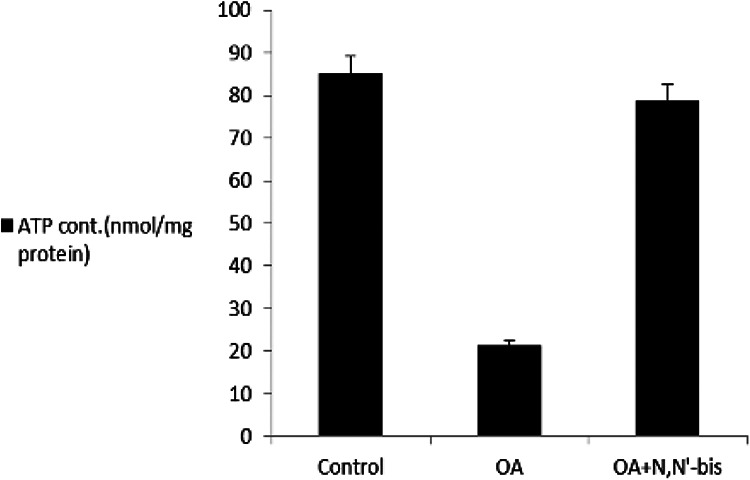
The efficacy of dpdo compound against ATP in the rat model of OA induced by MA. The data are presented as mean ± S. E. dpdo: *N*,*N*′-bis(1,5-dimethyl-2-phenyl-1,2-dihydro-3-oxopyrazol-4-yl) sebacamide; OA: osteoarthritis; MA: monoiodoacetate; SE: standard error.

The RF increased significantly by sevenfold in OA-induced rats as compared to control animals. Treatment of the animals with dpdo lowered the RF by 84.74% as compared to OA-treated rats. The cell viable number detected by SDH% is presented in Figure 9. The SDH was 92.02% in normal control animals and decreased significantly to 28.39% in OA-treated rats. The value of SDH was 90.52% in animals treated with dpdo. The baseline value of ROS for normal healthy rats was 7.25 ± 0.78 nm as shown in Figure 9. The arthritic rats presented higher ROS content than controls for mitochondria. More reduction in ROS contents was observed in the dpdo-treated group. The effects of dpdo on MMP (Δ*ψ*_m_) on day 45 are illustrated in Figure 10. The OA group had significantly increased MMP compared with the control healthy rats, had notably decreased by the combination of dpdo. These results suggested that dpdo was a remarkably enhanced collapse of MMP in OA rats. OA accelerated mitochondrial swelling as illustrated in Figure 11 where it was elevated as compared to control healthy rats. The swelling of different groups was diminished by 85.47% for dpdo. OA rats had less ATP content than control rats (Figure 12). The compound dpdo stimulated significantly more ATP content.

### Mitochondrial oxidative stress/antioxidant elevation

The levels of MDA in OA rats were found to be 5.2 times higher than in the normal control group (Table 2). This was accompanied by a marked reduction in the GSH level. In contrast, treatment with dpdo showed a significant lowering effect on MDA level than OA-treated rats. An elevation of GSH was also observed in dpdo in comparison with the OA group (Table 2). MPO and XO were elevated in OA rats as compared to control healthy animals (Table 2). Rats treated with dpdo significantly diminished the activities of MPO and XO of the OA-treated group. Thiol level declined in the OA group as compared to healthy rats (Table 2). Increment in thiol levels was observed in rats treated with dpdo.

**Table 2.** The efficacy of dpdo compound against oxidative/antioxidant status in the rat model of osteoarthritis induced by MA.^a^

Groups	GSH (µg mg^−1^ protein)	MDA (µg mg^−1^ protein)	MPO (nmol min^−1^ g^−1^)	XO (U g^−1^)	Thiol level (mmol g^−1^ tissue)
Control	0.77 ± 0.02	0.32 ± 0.01	22.72 ± 0.57	15.71 ± 0.5	16.16 ± 0.28
OA	0.38 ± 0.04^b^	1.67 ± 0.08^b^	33.29 ± 0.71^b^	31.19 ± 2.19^b^	8.21 ± 0.45^b^
OA and dpdo	0.71 ± 0.04^c,d^	0.43 ± 0.01^c,d^	23.46 ± 1.64^c^	19.53 ± 0.48^c,d^	15.04 ± 0.21^c,d^

dpdo: *N*,*N*′-bis(1,5-dimethyl-2-phenyl-1,2-dihydro-3-oxopyrazol-4-yl) sebacamide; OA: osteoarthritis; MA: monoiodoacetate; MDA: malondialdehyde; MPO: myeloperoxidase; XO: xanthine oxidase; SE: standard error.

^a^ The data are presented as mean ± SE.

^b^ The differences are significant in comparison with the control group (*p* ≤ 0.05).

^c^ Significantly different as compared to OA group.

^d^ Significantly different as compared to OA with *N*,*N*′-bis(1,5-dimethyl-2-phenyl-1,2-dihydro-3-oxopyrazol-4-yl)sebacamide.

### Histological sections and electron microscope evaluation

**Figure 13.**
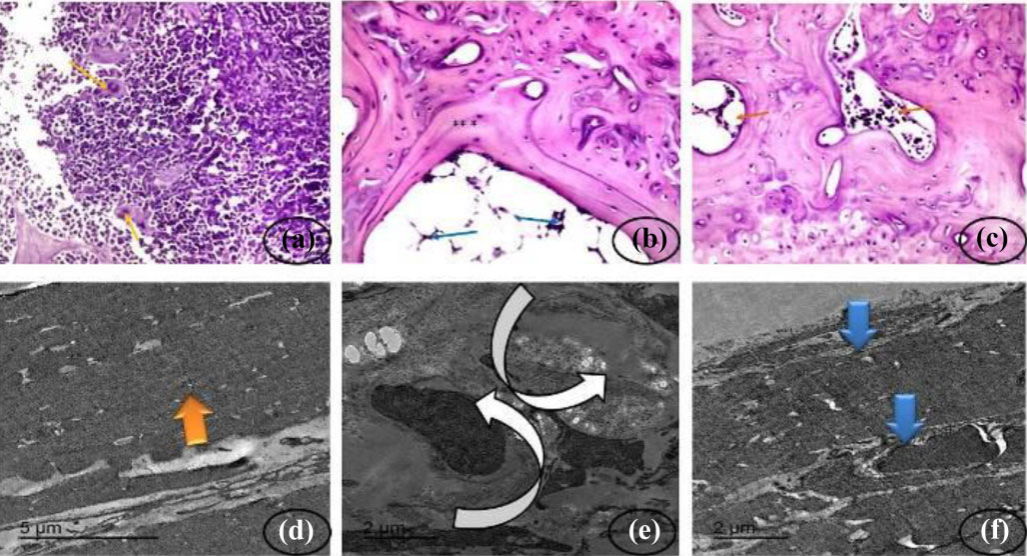
Histopathological slides of the bone cortex and bone marrow stained with hematoxylin and eosin in (a) control groups of rats showing normal bone cortex and normal bone marrow clearing normal cellularity of the bone marrow element (orange arrows) between normal bone trabeculae (×400). (b) OA control group showing highly degenerative bone marrow elements (blue arrows) with pyknotic osteoblasts, osteocytes (***) and more curvature cement line (×400); and (c) OA and dpdo compound showing amelioration and increasing in bone marrow elements and restoring compact structure (red arrows) (×400). TEM section of tissues obtained from the articular cartilage of the knee joints posttreatment with dpdo compound after induction of arthritis. Images showing collagen fibers of different treated groups. (d) Control group: showing intact compact cartilage fibers (orange arrow) with the intact articular septum (scale bar = 5 µm). (e) Osteoarthritic group (+ve control group): showing atrophy of cartilage fibers (rounded arrows) with distortion of articular septum (scale bar = 5 µm). (f) Osteoarthritic group + dpdo compound: showing restoration of most cartilage fibrils (blue arrows) with mostly normal chondrocyte size with intact compact cartilage fibrils and restoration of intra-articular septum (scale bar = 2 µm). TEM: transmission electron microscopic; dpdo: *N*,*N*′-bis(1,5-dimethyl-2-phenyl-1,2-dihydro-3-oxopyrazol-4-yl) sebacamide; OA: osteoarthritis.

Histopathological sections of the bone cortex and bone marrow showing control groups with normal bone cortex and normal bone marrow (Figure 13(a)). OA control group showing highly degenerative bone marrow elements with pyknotic osteoblasts and more curvature cement line (Figure 13(b)). OA and dpdo showing amelioration and increasing in bone marrow elements and restoring compact structure (Figure 13(c)). Furthermore, the present study was to identify whether dpdo improved articular cartilage degeneration and effects on OA (Figure 13). In the healthy control group, an intact structure of cells was identified with intact compact cartilage fibers and the intact articular septum (Figure 13(d)). Atrophy of cartilage fibers with distortion of the articular septum was observed in OA group (Figure 13(e)). The osteoarthritic group that treated with dpdo showed restoration of most cartilage fibrils with mostly normal chondrocyte size with intact compact cartilage fibrils and restoration of the intra-articular septum (Figure 13(f)).

### Comet images and mitochondrial

**Figure 14.**
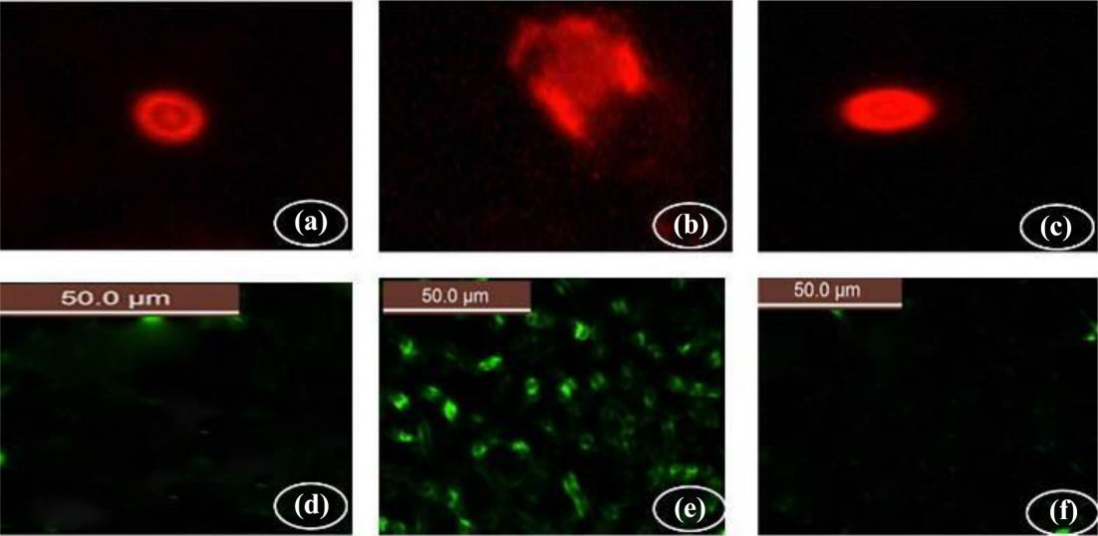
Comet images of cells derived from the knee joints tissues (a) control group showed intact nuclei and normal round cell without tail hallow. (b) OA group showed high degree of damage with appearance of highly damaged DNA with severe apoptotic cell with a large tail hallow in the form of a comet shape. (c) OA + dpdo compound group which showed more intact cells with less damaged DNA strands and less damaged nuclei. The fluorescent detection of mitochondrial membrane potential showed high improvement on the knee joint tissues-treated groups (d) control group showing non-accumulation of ROS in mitochondrial (50.0 µm). (e) AO-treated group showing high accumulation of ROS in the mitochondria as known that ROS accumulation is a hallmark of oxidative cell death (50.0 µm). (f) AO-treated groups with dpdo compound showing high amelioration in mitochondrial potential with very low or almost non-reactive oxygen species accumulation (50.0 µm). dpdo: *N*,*N*′-bis(1,5-dimethyl-2-phenyl-1,2-dihydro-3-oxopyrazol-4-yl) sebacamide; ROS: reactive oxygen species.

Comet images of cells derived from the knee joints tissues of the control group showed intact nuclei and normal round cell without tail hallow (Figure 14(a)). OA group showed high degree of damage with appearance of highly damaged DNA (Figure 14(b)). OA + dpdo group which showed more intact cells with less damaged DNA strands and less damaged nuclei (Figure 14(c)). The fluorescent detection of mitochondrial membrane potential showed high improvement on the knee joint tissues, control group showing non-accumulation of ROS in mitochondrial (50.0 µm) (Figure 14(d)). OA-treated group showing high accumulation of ROS in the mitochondria as known that ROS accumulation is a hallmark of oxidative cell death (50.0 µm) (Figure 13(e)). AO-treated groups with dpdo showing high amelioration in mitochondrial potential with very low or almost non-ROS accumulation (50.0 µm) (Figure 14(f)).

### Reverse transcriptase polymerase chain reaction

**Figure 15.**
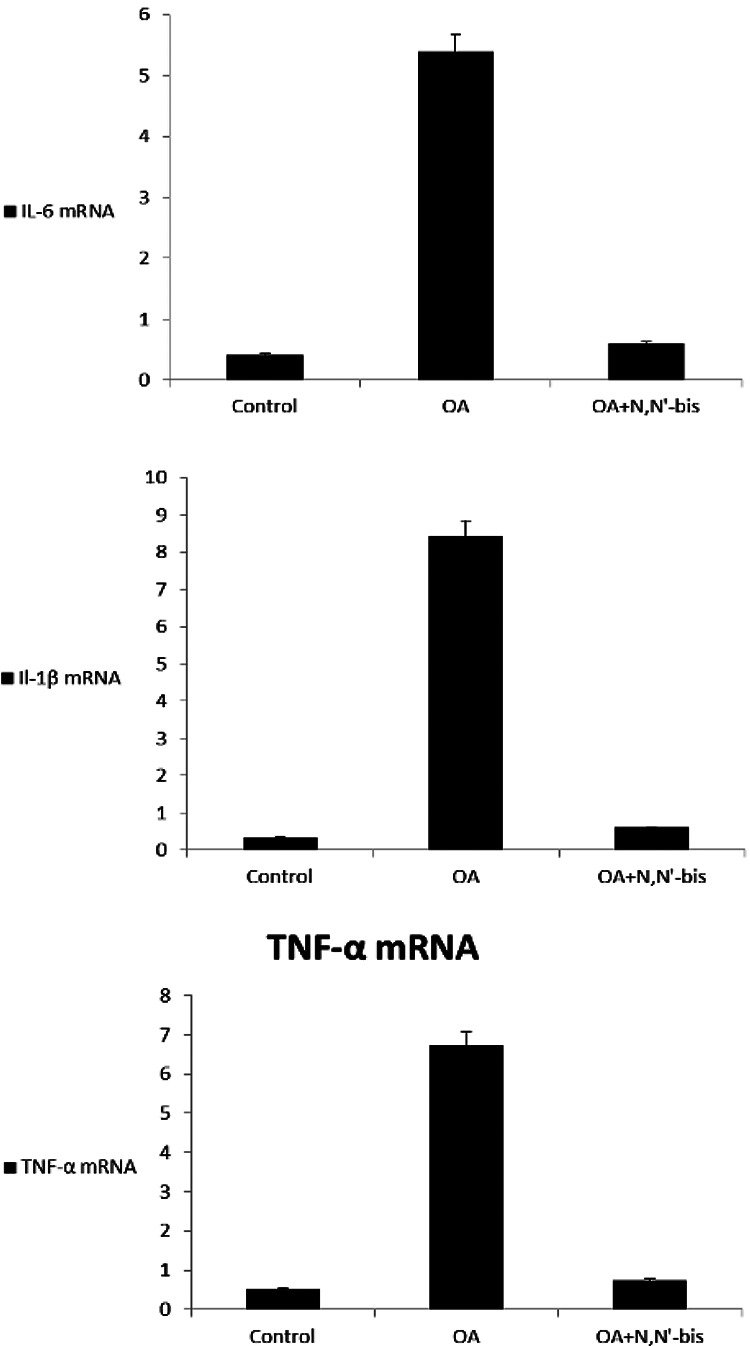
Statistical analysis of gene expressions of (IL-6, TNF-α, and IL-1β) in control and treatment with OA and dpdo compound by using quantitative reverse transcription polymerase chain reaction. IL: interleukin; TNF-α: tumor necrosis factor alpha; dpdo: *N*,*N*′-bis(1,5-dimethyl-2-phenyl-1,2-dihydro-3-oxopyrazol-4-yl) sebacamide.

RT-PCR analysis was conducted to investigate whether the inhibitory effect of dpdo on inflammation was due to the gene regulation of inflammatory mediators, like TNF-α, IL-6, and IL-1β as shown in Figure 15. The mRNA of TNF-α, IL-6, and IL-1β increased in OA-treated rats compared to those of the control group. In the combined group, OA with dpdo, the expression levels of TNF-α, IL-6, and IL-1β were markedly reduced as compared to streptozotocin (STZ) group as compared to the control group.

## Discussion

The MA model can rapidly produce OA lesions in rats, as compared to that found in the human disease. This model can be used for evaluating and studying possible OA drugs.^41^ The rat model for OA was used due to the facility of induction of arthritis in clear area and easily to be monitored without any changes takes place. All of the OA models described mimic human disease in some respects.^42^ Also, Yau and Holmdahl,^43^ demonstrated that animal models are optimal for studying OA, as environmental factors suspected to be of importance in human OA should be studied in a controlled way in animal models which can all present the immune system with a unique adjuvant exposure. Cartilage, which is considered as a tissue that is attached to the ends of bones in the joint, is devoid of blood vessels. Instead, it contains in a liquid that also acts as a lubricant. Within the soft tissues that produces this liquid, macrophages are found that have a dual purpose: to produce molecules that nourish and protect the cartilage tissues that protect the knee joints against an infection. Joint inflammation can develop and when that inflammation becomes chronic, macrophages produce molecules that degrade cartilage.^44^ OA is a rheumatic disease which is highly marked by the degradation of the articular cartilage, high mortality of chondrocytes, and inflammation of synovial joints.^45^ Several causes have been suggested for OA, including a hypothesis of oxidative stress.^46^ Excessive release of pro-inflammatory cytokines stimulates the level of reactive oxygen species (ROS) by stimulating neutrophils and activated macrophages, making them joint damage medium.^47^ Therefore, the accumulation of ROS occurred in the induced OA rats. In the present study, TNF-α, IL-6, CRP, RF, ROS, as well as all the mitochondrial markers such as MMP, swelling mitochondria, cytochrome *c* oxidase, and serum oxidative/antioxidant status (MDA level and activities of MPO and XO) were elevated significantly in OA rats as compared to control healthy animals. However, the activity of SDH, levels of ATP, GSH, and thiol were markedly diminished in the OA group compared to normal control rats.

Pro-inflammatory cytokines are multifunctional proteins that play a vital role in the cellular communication. TNF-α is one of the most important pro-inflammatory cytokines and plays a critical part in immune disorder pathogenesis. TNF-α controls a wide range of pathological processes, including cellular inflammation, swelling, differentiation, and death.^48^ IL-6 and TNF-α have critical roles in modulating tissue inflammation in OA; moreover, they can reflect the severity of inflammation in vivo.^48^ They are known to increase expression that induces catabolic events as they enhance MMP.^49^ In the present study, IL-6 and TNF-α were elevated in OA rats. TNF-α downregulates the synthesis of extracellular matrix components by inhibiting chondrocyte anabolic activity of and decreasing type II collagen production.^49^ There is growing proof that in the inflammatory process, CRP has a vital function and its level rises in circulation.^50^ In reaction to the increased level of IL-6, transcriptional induction of the *CRP* gene happens primarily in liver cells. There is sufficient evidence that during OA the cartilage is damaged.^51^ The two major pro-inflammatory cytokines, IL-6 and TNF-α, have been associated with the prevalence of joint space narrowing and prediction of knee cartilage loss.^52^ It could be explained that IL-6 and TNF-α influence synovial hyperplasia progression, leading to OA growth and advancement.^44^ Our finding is in agreement with the findings of Wojdasiewicz et al.^52^ who found that TNF-α level was increased in OA joint tissues and synovial fluid compared with normal individuals. Moreover, it has been recognized that RF plays an important role in the differential diagnosis of polyarthritis because it makes it possible to identify rheumatoid patients.^53^ It was also used to predict the response of TNF-α. In the present study, there is a direct relationship between the RF and TNF-α. During OA, neutrophil degradation results in the discharge of enzymes leading to oxidative stress and respiratory bursts.^54^ Thus, increased MPO in inflamed rheumatoid arthitis (RA) cartilage is connected with the persistent development of disease.^55^ In mitochondrial aerobic respiration, the electron transport is linked via the inner mitochondrial membrane throughout four enzyme complexes (I–IV) with complex V (ATP synthase) from ADP.^56^ In the present study, in healthy and OA rats, mitochondrial function was evaluated by analyzing some complex enzymes of the respiratory chain II, IV, and Δ*Ψ*_m_. The mitochondrial dysfunction in the OA shows decreased activity of complex II compared to normal healthy rats, as mentioned before by Blanco et al.^57^ Such mitochondrial impairment can affect numerous pathways involved in cartilage degradation, oxidative stress, defective chondrocyte biosynthesis, and growth responses. In addition to mitochondrial dysfunction, increased cytokine-induced chondrocyte inflammation and matrix catabolism, cartilage matrix calcification, and increased chondrocyte apoptosis have been observed.^50^ The mitochondria respiratory chain integrity is important for producing ATP and maintaining the Δ*Ψ*_m_.^58^ Since mitochondrial depolarization in itself accounts for depletion of ATP in chondrocytes, the Δ*Ψ*_m_ in OA chondrocytes were evaluated in the current investigation. MMP (Δ*Ψ*_m_) is a factor that determines the viability of mitochondria involved in the elimination process of damaged mitochondria.^59^ The collapsing of Δ*Ψ*_m_ is correlated with mitochondrial swelling, disruption of the outer mitochondrial membrane, and the liberation from the intermembrane space of proapoptotic factors such as cytochrome *c*, apoptosis-inducing factor, and procaspases. All of these results are consistent with the findings of present data and several other published studies that showed more apoptotic chondrocytes in OA than in normal cartilage.^60^ Pro-inflammatory cytokines, including IL-6, IL-17, and TNF-α in OA, cause catabolic activity. Increased level of inducible NO biosynthesis in OA chondrocytes results in accumulated NO, which suppresses proteoglycan and collagen synthesis in chondrocytes^61^ and mediates the induction of matrix-degrading MMPs by accelerating the catabolic cascade induced by IL-1β or TNF-α. cytochrome *c* is released from mitochondria to the cytosol during apoptosis to trigger a caspase cascade that connects the cell to the cycle of death. It was suggested that the swelling of the mitochondrial matrix triggered by the apoptotic stimuli causes the discharge of cytochrome *c*.^58^ This result is in parallel with the observation in the current study where the cytochrome *c* increased in OA rats. The elevation of the MPO activity is one of the best diagnostic tools of oxidative stress and inflammatory biomarkers in rheumatoid arthritis and other chronic diseases.^62^ Hamza and Diab^63^ reported that oxidative stress plays a key role in the release of MPO from these cells. MPO generates reactive oxidants and other free radicals either through its process of peroxidation or through a process of halogenation, depending on the accessibility of the substrate.^64^ The main reason for OA could be the excess accumulation of ROS, causing an increase in the LPO level.^65^ In the present study, MDA levels elevated that became an indicator for the cellular membrane damage. The antioxidant enzymes superoxide dismutase (SOD) and catalase (CAT)) were decreased because they regulated superoxide anion and H_2_O_2_. Moreover, endogenous antioxidants GSH and thiol were decreased in the induced OA group. Current therapy alternatives for OA concentrate on pain reduction (non-steroidal anti-inflammatory drugs (NSAIDs)) and joint visco-supplementation (hyaluronic acid intra-articular injections). In addition to having a short-term impact, chronic use of some of these drugs, particularly NSAIDs, can trigger severe adverse events, including poisoning and thromboembolism risk.^49^ Moreover, NSAIDs increased the risk of serious gastrointestinal complications when administered through the oral route.^64^ This finding supported the current finding by using safe anti-inflammatory agent against COVID-19 with no common side effects of NSAIDs.

Multicomponent and green synthetic route for the synthesis of pyrazole derivatives as antioxidants and antimicrobial agents was reported by Ambethkar et al.^66^ The most important aspect of using pyrazole derivatives is that a lot of pyrazole derivatives are acknowledged to possess a wide range of antibacterial bioactivities^22^ and act as DNA damages inhibitors. Gomez et al.^67^ synthesized a series of novel pyrazole derivatives as DNA injury inhibitors that exhibit antibacterial activities against some gram-negative and gram-positive (such as *Streptococcus pneumoniae*) bacterial strains and this point supported greatly our suggestion by success of the novel synthesized pyrazole against severe pneumonia occurred by COVID-19 and thus we think that these derivatives will play a great role against severe infection occurred by COVID-19. The remarkable chemical diversity of heterocyclic compounds continues to be of relevance to drug discovery.^68^ Drug development has been a chief component in the rapid maturation of the field of medicinal chemistry during the past several decades; during this period, the scientific community has paid significant attention to the development of challenging molecular architectures of nitrogen-containing heterocyclic compounds such as pyrazole. There is a growing body of evidence that pyrazole and its derivatives provide a viable and valuable area for drug discovery. Here, we have presented an overview of a highly functionalized pyrazole derivatives and with a broad range of its biological activities that can optimally present a way to disinfect severe pneumonia infections that could be induced by COVID-19. The ability to predict drug-like properties along with recent technological advances could be sufficient to revitalize the exploitation of the value of pyrazole derivatives in the quest for new drugs especially for the viral infections like COVID-19.

In conclusion, the alterations caused by OA which represent an imbalanced situation that contributes to the energy metabolism and immune response of the OA disease. Δ*ψ*_m_ is considered as an early indicator of cellular apoptosis. The compound dpdo can have therapeutic benefits through reduced oxidative stress and suppression of the level of inflammatory injury in OA-animals. These findings suggest that the therapeutic potential of dpdo for the treatment of autoimmune diseases combined through the mitochondrial pathway without causing side effects as the other standard therapy. The current data were confirmed by cytokine estimation, histological and electron microscope examination.

## Supplemental material

Supplemental_material - Synthesis of *N*,*N*′-bis(1,5-dimethyl-2-phenyl-1,2-dihydro-3-oxopyrazol-4-yl) sebacamide that ameliorate osteoarthritis symptoms and improve bone marrow matrix structure and cartilage alterations induced by monoiodoacetate in the rat model: “Suggested potent anti-inflammatory agent against COVID-19” Click here for additional data file. Supplemental_material for Synthesis of *N*,*N*′-bis(1,5-dimethyl-2-phenyl-1,2-dihydro-3-oxopyrazol-4-yl) sebacamide that ameliorate osteoarthritis symptoms and improve bone marrow matrix structure and cartilage alterations induced by monoiodoacetate in the rat model: “Suggested potent anti-inflammatory agent against COVID-19” by MS Refat, RZ Hamza, AMA Adam, HA Saad, AA Gobouri, FA Al-Salmi, T Altalhi and SM El-Megharbel in Human & Experimental Toxicology
