# Supplementary material for: Synthesis of N,N′-bis(1,5-dimethyl-2-phenyl-1,2-dihydro-3-oxopyrazol-4-yl) sebacamide that ameliorate osteoarthritis symptoms and improve bone marrow matrix structure and cartilage alterations induced by monoiodoacetate in the rat model: “Suggested potent anti-inflammatory agent against COVID-19”
Source: Hum Exp Toxicol. 2020 Aug 25;40(2):325–41. doi: 10.1177/0960327120945779 (PMC7447864; doi:10.1177/0960327120945779)
Supplement: Supplemental_material - Synthesis of N,N′-bis(1,5-dimethyl-2-phenyl-1,2-dihydro-3-oxopyrazol-4-yl) sebacamide that ameliorate osteoarthritis symptoms and improve bone marrow matrix structure and cartilage alterations induced by monoiodoacetate in the rat model: “Suggested potent anti-inflammatory ag [file Supplemental_material.pdf]

Synthesis of N,N'-bis(1,5-dimethyl-2-phenyl-1,2-dihydro-3-oxopyrazol-4-yl) sebacamide that ameliorate osteoarthritis symptoms and improve bone marrow matrix structure and cartilage alterations induced by monoiodoacetate in the rat model

Moamen S. Refat, Reham Z. Hamza, A.M.A. Adam, H.A. Saad, Adil A. Gobouri, Fawziah A. Al-Salmi, T. Altalhi, Samy M. El-Megharbel

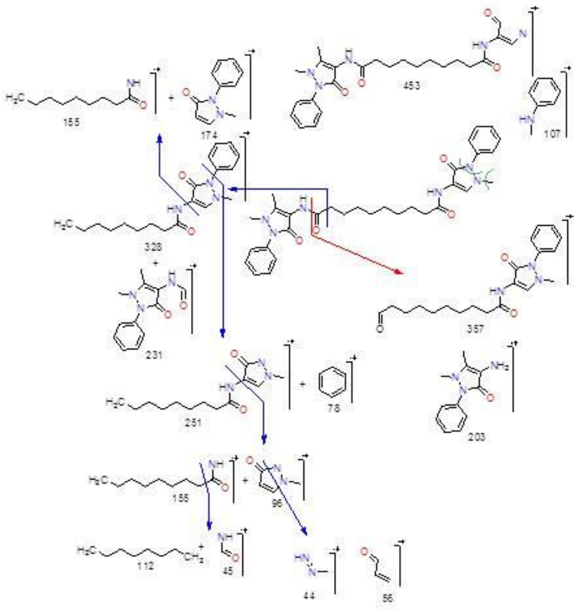

Mass fragmentations of dpdo compound.

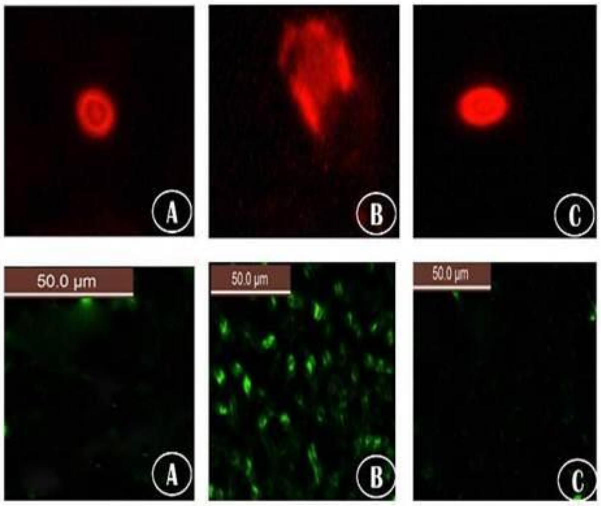

Comet images of cells derived from the knee joints tissues of different treated groups showing ameliorative effect of the synthesized compound
